# News and views: AUTOTACs join the arena for targeted protein degradation

**DOI:** 10.1007/s00204-022-03312-3

**Published:** 2022-05-17

**Authors:** Andreas O. Mieland, Mandy Beyer, Oliver H. Krämer

**Affiliations:** Institute of Toxicology, Mainz University Medical Center, Mainz, Germany

A better treatment of human diseases requires increased knowledge on the dysregulated proteins that cause them. Some of these proteins can be targeted with drugs that activate lost signal pathways or repress overactive functions that disrupt homeostasis. However, for several diseases there are no direct inhibitors of rogue proteins or existing drugs are not effective. The last years faced the advent of proteolysis targeting chimeras (PROTACs). These heterobifunctional molecules consist of ligands that attract E3 ubiquitin ligases and inhibitors that avidly bind proteins. This consequently triggers their ubiquitin-dependent proteasomal degradation. Due to a lack of a general E3 ubiquitin ligase that promotes poly-ubiquitinylation, there is no universally applicable tool for PROTAC development. Moreover, the pore size of proteasomes is 13 Å, which does not permit the elimination of aggregated pathogenic proteins by the ubiquitin–proteasome-system (UPS). Ji et al. (2022) recently published a proof-of-concept study for a more promiscuous approach of protein degradation. Their strategy exploits macroautophagy (hereafter autophagy) as pathway to eliminate unwanted and harmful monomeric and aggregated proteins. This lysosomal protein degradation and recycling pathway acts in parallel to the UPS (Limpert et al. 2018). Based on their previous works, structural modeling, and structure–activity relationship studies, the groups around Kim, Kim, and Kwon developed autophagy-targeting chimeras (AUTOTACs) as new degrader-type drugs (Ji et al. 2022). These multifunctional agents are based on autophagy-targeting ligands (ATLs) which bind to the ZZ domain of the major autophagy cargo receptor p62. This activates its self-oligomerization and promotes the formation of catabolic autophagosomes. ATLs remotely resemble the N-terminal arginine moiety in arginylated proteins, which is a preferred binding structure for p62 (Ji et al. 2022). Chemical coupling of target-binding ligands (TBLs) to ATLs yielded fusion molecules that were named AUTOTACs. As TBLs, these authors used antagonistic ligands for estrogen receptor beta (ERβ), androgen receptor (AR), and methionine aminopeptidase-2 (MetAP2). The resulting AUTOTACs turned out to be nanomolar inducers of the degradation of ER*β*, AR, and MetAP2 by autophagy. Micromolar doses of AUTOTACs inhibited the proliferation of human cancer cell lines. Neither the p62-binding ATL nor the TBL alone induced these beneficial effects, which were ubiquitin-independent and even increased upon inhibition of the UPS. Furthermore, the authors elegantly proved a functional involvement of p62 and the autophagy machinery with a mutant p62 molecule lacking an intact ZZ domain, RNAi-based techniques, and knockout cells. Curiously, while biochemical AR-dependent signaling was similarly blocked by the AR antagonist and the AR-AUTOTAC, only the AR-AUTOTAC inhibited cell growth (Ji et al. 2022). This shows that a combined AR inhibition and autophagy induction has benefits beyond AR inactivation. Despite such hopes, careful research is required to see how AUTOTACs affect the outcome of classical chemotherapy with drugs inducing DNA replication stress and DNA damage. Autophagy induction may blunt their cytotoxic anti-tumoral effects (Limpert et al. 2018).

The study by Ji, Kim, Lee, and colleagues additionally suggests an unprecedented therapeutic use of AUTOTACs for diseases that are caused by misfolded proteinaceous aggregates. These cannot be eliminated by the UPS and are not effectively cleared through autophagy. When a chemical chaperone that binds exposed hydrophobic protein patches, such as 4-phenylbutyric acid (PBA), was used as TBL bound to an ATL, misfolded ubiquitin-conjugated proteins were targeted to autolysosomes for degradation. This was found with mutant aggregate-forming proteins in human cells (desmin, huntingtin, tau) and in a mouse Alzheimer dementia model with a mutated, aggregation-prone tau protein. Like for the proto-oncogenes, the ATL and PBA fragments alone did not trigger the elimination of protein aggregates. Notably, AUTOTACs did not remove properly folded wild-type proteins that are not aggregation-prone. A further pharmacologically remarkable feature of AUTOTACs is their apparent recycling from the lysosome-autophagy pathway, enabling multiple rounds of sustained protein degradation (Ji et al. 2022). PBA is a carboxylic acid-based histone deacetylase inhibitor and produces beneficial effects in neuropathies. However, since the carboxylic acid function was esterified in the PBA-AUTOTACs, one can exclude histone deacetylase inhibition as mechanism of action of PBA-AUTOTACs. Furthermore, these effectively eliminated aggregated proteins at micromolar doses while carboxylic acids are millimolar histone deacetylase inhibitors (Krämer et al. 2001).

In summary, AUTOTACs are innovative, interesting, and promising molecules which offer several advantages over existing strategies.
